# Reproductive performance of resident and migrant males, females and pairs in a partially migratory bird

**DOI:** 10.1111/1365-2656.12691

**Published:** 2017-06-19

**Authors:** Hannah Grist, Francis Daunt, Sarah Wanless, Sarah J. Burthe, Mark A. Newell, Mike P. Harris, Jane M. Reid

**Affiliations:** ^1^ Institute of Biological and Environmental Sciences School of Biological Sciences University of Aberdeen Aberdeen UK; ^2^ Centre for Ecology & Hydrology Bush Estate Midlothian UK; ^3^ Scottish Ornithologists' Club Waterston House Aberlady UK; ^4^ Scottish Association for Marine Science Argyll UK

**Keywords:** carry‐over effects, demography, European shag, fitness, phenology, population structure, seabird

## Abstract

1. Quantifying among‐individual variation in life‐history strategies, and associated variation in reproductive performance and resulting demographic structure, is key to understanding and predicting population dynamics and life‐history evolution. Partial migration, where populations comprise a mixture of resident and seasonally migrant individuals, constitutes a dimension of life‐history variation that could be associated with substantial variation in reproductive performance. However, such variation has rarely been quantified due to the challenge of measuring reproduction and migration across a sufficient number of seasonally mobile males and females.
2. We used intensive winter (non‐breeding season) resightings of colour‐ringed adult European shags (*Phalacrocorax aristotelis*) from a known breeding colony to identify resident and migrant individuals. We tested whether two aspects of annual reproductive performance, brood hatch date and breeding success, differed between resident and migrant males, females and breeding pairs observed across three consecutive winters and breeding seasons.
3. The sex ratios of observed resident and migrant shags did not significantly differ from each other or from 1:1, suggesting that both sexes are partially migratory and that migration was not sex‐biased across surveyed areas.
4. Individual resident males and females hatched their broods 6 days earlier and fledged 0.2 more chicks per year than migrant males and females on average. Resident individuals of both sexes therefore had higher breeding success than migrants.
5. Hatch date and breeding success also varied with a pair's joint migratory strategy such that resident–resident pairs hatched their broods 12 days earlier than migrant–migrant pairs, and fledged 0.7 more chicks per year on average. However, there was no evidence of assortative pairing with respect to migratory strategy: observed frequencies of migrant–migrant and resident–resident pairs did not differ from those expected given random pairing.
6. These data demonstrate substantial variation in two key aspects of reproductive performance associated with the migratory strategies of males, females and breeding pairs within a partially migratory population. These patterns could reflect direct and/or indirect mechanisms, but imply that individual variation in migratory strategy and variation in pairing among residents and migrants could influence selection on migration and drive complex population and evolutionary dynamics.

## Graphical abstract

The authors use breeding observations and winter resightings of a partially migratory European shag population to show that residents hatch broods earlier and fledge more chicks than migrants, and importantly that overall reproductive performance is strongly related to the combination of migratory strategies within breeding pairs.

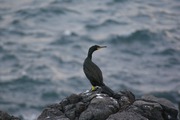

## 1 INTRODUCTION

Quantifying variation in life‐history strategies among different categories of individuals within any population, and associated variation in individual fitness and population‐level demographic structure, is key to understanding and predicting population and evolutionary dynamics (Gillis, Green, Middleton, & Morrissey, 2008; Reid et al., 2010; Vindenes, Engen, & Sæther, 2008). Partial migration, where some individuals within a population migrate between locations across seasons while other individuals from the same population remain resident at a single location, constitutes a major form of life‐history variation that occurs widely, including in many species of fish, amphibians, mammals and birds (e.g. Boyle, 2008; Brodersen, Nilsson, Hansson, Skov, & Brönmark, 2008; Chapman, Brönmark, Nilsson, & Hansson, 2011; Grayson & Wilbur, 2009; Hebblewhite & Merrill, 2011; Hegemann, Marra, & Tieleman, 2015). Such among‐individual variation in “migratory strategy,” most simply defined as migrant vs. resident (e.g. Bai, Severinghaus, & Philippart, 2012; Gillis et al., 2008), results in individuals that co‐exist during either the breeding or non‐breeding season experiencing geographically and ecologically disparate environments during the other season (Boyle, 2008; Chapman et al., 2011; Grayson & McLeod, 2009). Among‐individual variation in migratory strategy might therefore be associated with among‐individual variation in key fitness components (i.e. survival and/or reproductive performance), creating substantial structured variation in demography (Gillis et al., 2008; Kokko, 2011).

Such demographic structure could directly affect population dynamics, and also create selection on migration. If migratory strategy were to some degree repeatable within individuals across years and associated with differences in survival, selection could cause the frequencies of migrants and residents to change across years and age‐classes within generations (i.e. “selective disappearance,” e.g. van de Pol & Verhulst, 2006). Furthermore, if migratory strategy were heritable, selection would drive evolutionary change across generations (Boyle, 2008; Kaitala, Kaitala, & Lundberg, 1993; Lundberg, 1987; Pulido, 2007). Selection and the resulting demographic and evolutionary responses could be direct if migratory strategy itself affects fitness components, or indirect if individual migratory strategy and fitness components are both intrinsically affected by some other temporary or permanent individual trait or state. Resulting structured changes in the frequencies of migrants vs. residents across years and generations could then affect population‐wide reproduction, survival and spatial structure (Adriaensen & Dhondt, 1990; Sanz‐Aguilar, Bechet, Germain, Johnson, & Pradel, 2012), thereby further altering overall population growth rate and spatio‐temporal dynamics.

In sexually reproducing species, such population dynamic and evolutionary consequences of partial migration will also depend on the degree to which females vs. males migrate or remain resident, and on the degree to which relationships between migratory strategy and key fitness components are consistent or different in the two sexes. Partial migration can be substantially sex‐biased with one sex being much more likely to migrate than the other, as observed in newts (Bloch & Grayson, 2010; Grayson & McLeod, 2009) and some birds (Boyle, 2008; Bai et al., 2012; Table S1). In other systems, including some birds and mammals, both sexes are partially migratory to similar degrees (Hebblewhite & Merrill, 2011; Warriner, Warriner, Page, & Stenzel, 1986; Table S1). However, relationships between fitness components and migratory strategy could still be sex‐specific in such systems, for example stemming from sexual dimorphism in body size and associated tolerance to environmental harshness, or from sex‐specific determinants of reproductive success that might depend on migration, such as territory acquisition or body condition (Bai et al., 2012; Chapman et al., 2011; Jahn, Levey, Hostetler, & Mamani, 2010).

Furthermore, in populations where both sexes are partially migratory, reproductive performance might vary with the combined migratory strategy of a breeding pair, rather than with the strategies of females and males independently (e.g. Brommer, Karell, Aaltonen, Ahola, & Karstinen, 2015). A key assumption of many models of partial migration is that individuals that remain resident in breeding areas can pre‐emptively occupy high‐quality territories before migrant individuals return, and consequently attain higher reproductive success (Griswold, Taylor, & Norris 2010; Kokko, 2011). However, the potential reproductive advantage that one pair member attains by remaining resident might be reduced if its mate is a migrant and returns late. Resident–resident pairs may therefore have substantially higher reproductive success than migrant–migrant pairs or pairs with mixed migratory strategies, depending on system‐ and sex‐specific costs, benefits and underlying causes of migration. Assortative pairing with respect to migratory strategy might also arise. In particular, frequencies of resident–resident and migrant–migrant pairs might exceed those expected given random pairing if individuals are more likely to form pairs with those that arrive at similar times (e.g. Anderson, Novak, Smith, Steenhof, & Heath, 2015; Gunnarsson, Gill, Sigurbjörnsson, & Sutherland, 2004). Such assortative pairing could increase the population‐wide variance in reproductive success, increase selection on migration (Pulido, 2007), and even facilitate sympatric reproductive isolation (Bearhop et al., 2005; Rolshausen, Segelbacher, Hermes, Hobson, & Schaefer, 2013).

Understanding the population dynamic and evolutionary consequences of partial migration therefore requires studies that quantify relationships between individual male, female and pair migratory strategies and reproductive performance. However, collecting sufficient data is challenging, requiring numerous individuals of both sexes to be tracked across different seasons and geographical locations in order to record migratory strategy and reproductive performance. To date, relatively few studies have related aspects of reproductive performance to individual migratory strategy in partially migratory systems, and these studies report mixed results (Table S1). Residents can show better reproductive performance than migrants (Adriaensen & Dhondt, 1990; Anderson et al., 2015; Warriner et al., 1986), as commonly assumed by evolutionary models of partial migration (Kaitala et al., 1993; Kokko, 2011). However, this pattern is not consistent across all systems or aspects of reproductive performance, such as breeding probability and timing, number of offspring, offspring condition and juvenile survival (e.g. Grayson & McLeod, 2009; Hebblewhite & Merrill, 2011; Table S1).

The few studies that have investigated relationships between individual migratory strategy and reproductive performance in both sexes within a single population show that effects can be sex‐specific (Table S1). For example, resident males paired earlier than migrant males in snowy plovers (*Charadrius nivosus*) but there was no such relationship for females (Warriner et al., 1986). Conversely, resident female Lanyu scops owls (*Otus elegans*) were more likely to nest successfully than migrant females but there was no such relationship for males (Bai et al., 2012). Only one study has quantified relationships between pair (rather than individual) migratory strategy and reproductive performance in a partially migratory population. Warkentin, James, and Oliphant (1990) showed that the annual breeding success of “resident male, migrant female” merlin (*Falco columbarius*) pairs exceeded that of “resident female, migrant male” pairs (4.8 ± 0.1 *SE* vs. 4.3 ± 0.2 *SE* chicks), and was substantially higher than “migrant–migrant” pairs (4.0 ± 0.1 *SE* chicks). However, the small sample sizes available for “resident–resident” pairs precluded rigorous statistical comparison. Therefore, to understand the potential consequences of within‐population variation in migratory strategy and associated variation in reproductive performance for population dynamics and evolution, we require studies that quantify relationships between migratory strategy and reproductive performance in males and females separately, and across combined breeding pairs.

We used intensive nest monitoring and winter colour‐ring resightings from partially migratory European shags (*Phalacrocorax aristotelis*; hereafter “shag”) to quantify variation in key aspects of reproductive performance, hatch date and breeding success, in relation to migratory strategies of individual males and females, and of breeding pairs. First, across individuals that were resighted in winter and hence classified as resident or migrant, we estimated the degree of partial migration in each sex across the surveyed areas and tested whether males or females were more likely to be migratory. Second, we tested whether hatch date or breeding success varied with migratory strategy in males and females separately. Third, we tested whether these aspects of reproductive performance varied with pair migratory strategy. Finally, we tested whether pairing was assortative with respect to migratory strategy. We thereby quantified key relationships between male and female migratory strategy and reproductive performance, and discuss the possible causes of these relationships and their consequences for the dynamics of partially migratory populations and associated life‐history evolution.

## 2 MATERIALS AND METHODS

### 2.1 Study system

European shags are large, diving seabirds that inhabit rocky coastlines in western Europe (Wanless & Harris, 1997). Shags provide a useful system to relate reproductive performance to migratory strategy because they breed in large colonies where aspects of reproduction such as hatch date and breeding success can be readily recorded. Furthermore, adult shags breeding at a single colony can occupy a range of non‐breeding (winter) locations, including the breeding colony, and are therefore partially migratory (Sponza, Cosolo, & Kralj, 2013; Grist et al., 2014).

A breeding colony of shags on the Isle of May National Nature Reserve, Firth of Forth, Scotland (56°11′N, 2°33′W) has been the focus of an intensive long‐term demographic study (Aebischer, Potts, & Coulson, 1995; Daunt, Wanless, Harris, & Monaghan, 1999; Frederiksen, Daunt, Harris, & Wanless, 2008; Harris, Buckland, Russell, & Wanless, 1994). During 1997–2012, chicks hatched across the colony were ringed with a British Trust for Ornithology (BTO) metal ring and a coloured plastic ring engraved with a unique three letter code. Previously unringed or BTO‐ringed adults were captured at their nests and colour‐ringed. Colour rings can be read from up to 150 m through a telescope or digital camera, allowing ringed individuals to be identified from field resightings without recapture (Grist et al., 2014).

Clutches are typically laid during April to June, and pairs rear 0–4 chicks per year in a single brood (Wanless & Harris, 1997). There is therefore considerable within‐year variation in hatch date and breeding success, with higher breeding success associated with earlier breeding (Aebischer, 1993; Daunt et al., 1999). Ring resighting and recovery data show that shags typically breed first at 3 years of age (Aebischer et al., 1995), and that annual adult survival probability is typically ≥0.85 (excluding “wreck” winters with high mortality, Frederiksen et al., 2008). Adults therefore generally survive to breed in multiple years. On average, *c*. 50% of surviving adults change mates between years (Aebischer et al., 1995), but breeding dispersal among colonies is rare (Barlow, Daunt, Wanless, & Reid, 2013). Adults' reproductive timing and success can therefore be directly recorded with little error and few missing data (see *Statistical analyses*).

### 2.2 Winter location

Shags have a partially wettable plumage and must return to land every day, restricting their year‐round distribution to suitable coastal habitat (Grémillet, Tuschy, & Kierspel, 1998; Rijke, 1968). Colour‐ringed shags can therefore be observed in winter, both at night roosts where individuals congregate at dusk (typically on cliffs and islands), and at day roosts where individuals periodically rest between diving bouts (including on rocks and harbour walls).

**Figure 1:**
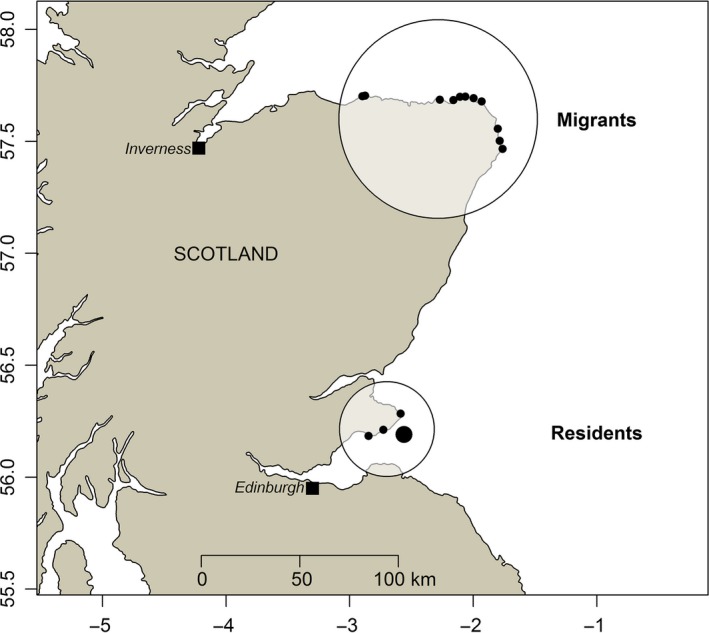
Locations of resighting surveys for colour‐ringed shags during winters 2009–2012. Circles identify migrant and resident wintering areas, and points are known roost locations within each area. The larger point marks the Isle of May breeding colony [Colour figure can be viewed at http://www.wileyonlinelibrary.com]

To identify winter locations of shags that breed on the Isle of May and hence identify samples of migrant and resident individuals, extensive colour‐ring resighting surveys were undertaken during three winters (2009–2010, 2010–2011 and 2011–2012). Full survey methods are described in Grist et al. (2014). For current analyses, both night and day roosts that are known to be used by shags that breed on the Isle of May were surveyed approximately every 1–2 weeks through each winter (September–February). The surveyed areas comprised the Isle of May night roost and its adjacent day roosts, and roosts across north‐east Scotland (Figure 1). During each survey, experienced observers identified colour‐ringed shags using a 60× magnification telescope (Grist et al., 2014). Surveys were strategically timed to maximise expected site‐specific resighting efficiency as influenced by weather, time of day, tide and sea state, and lasted 30–300 min depending on viewing conditions and the number and turnover of shags present. These surveys were designed to identify samples of resident individuals that remained at the Isle of May year‐round and samples of migrant individuals that wintered at key sites elsewhere (Figure 1), but not to explicitly estimate the overall population‐wide proportions of residents vs. migrants across all winter locations. As colour‐ringed individuals can only be resighted when they are on land, the probability of resighting any individual that is present in an area during any single survey is relatively low, particularly during mid‐winter when individuals can spend over 90% of daylight hours foraging (Daunt, Afanasyev, Silk, & Wanless, 2006; Daunt et al., 2014; Lewis, Phillips, Burthe, Wanless, & Daunt, 2015). However, the repeated surveys undertaken throughout the winter increased the probability that individuals that were present would be resighted on one or multiple occasions (Appendix S1).

Previous analyses of these data established that the distances from the Isle of May at which individual shags were resighted were highly repeatable within winters (mid‐winter repeatability >0.88, Grist et al., 2014), showing that individuals typically remain in a single area throughout winter. Consequently, for current analyses, individuals were classified as resident or migrant based on resightings spanning 6 October–10 February within each winter (hereafter “winter period”). This period encompasses the time for which most migrants were away from the Isle of May (Grist et al., 2014). However, despite this restriction, some individuals could still have been resighted on the Isle of May before migrating or after returning, causing individuals that had migrated to unsurveyed locations to be misclassified as residents. To minimise any such misclassification, only individuals that were resighted at least twice on the Isle of May or associated day roosts at least 7 days apart within the focal winter period were classified as residents, and all individuals that were resighted at least once away from the Isle of May area were classified as migrants. Any remaining misclassification of migrants as residents is therefore likely to be minimal, and would render any estimated difference in reproductive performance conservative. Furthermore, across all three focal winters, there were only 14 observed transitions of individuals between the Isle of May and focal migrant areas within winters, resulting from seven observed mid‐winter return movements (as distinct from individuals' early‐winter outward and late‐winter return migration movements). The mid‐winter transitions involved seven out of the total of 295 (2.4%) assigned migrant individuals, and 14 out of the total of 1,620 (<1%) potential transitions between consecutive resightings of an individual. These data confirm that individuals can be categorised as residents or migrants within any focal winter with little uncertainty (Appendix S1, see also Grist et al., 2014).

Previous analyses also showed that individual adult shags are resighted at highly repeatable distances from the Isle of May across different winters as well as within winters (across‐year mid‐winter repeatability ≥0.73, Grist et al., 2014). These data show that most individual adults are consistently resident or migrant across winters, as further evidenced by data collected up to 2015 (see *Discussion*). However, in case individuals did switch strategy between our focal winters, current analyses were restricted to observations of reproductive performance of individual adults that bred on the Isle of May in at least one summer during 2010–2012 that had been resighted and classified as resident or migrant during the immediately preceding winter (i.e. utilising data from adjacent winter–summer periods only). The spatio‐temporal distribution of survey effort varied among the three winters, due to variation in environmental conditions and observer availability (Appendix S1). In particular, there were fewer mid‐winter surveys of the Isle of May in winter 2009–2010. Consequently, among‐winter variation in the numbers and proportions of shags that were classified as residents and migrants probably predominantly reflects variation in survey effort rather than in shag migration ecology. Such variation does not impede our current aim of comparing reproductive performance between samples of resident and migrant males and females.

### 2.3 Reproductive performance

To quantify reproductive performance, almost all shag nest sites on the Isle of May were monitored intensively during March–August 2010, 2011 and 2012. The nest locations of all colour‐ringed breeding adults were recorded, and males were distinguished from females by larger body size and croaking call (Snow, 1963). A breeding attempt was defined as occurring when a fully built nest, eggs or chicks were observed.

All chicks that survived to approximately 20 days post‐hatch were ringed and wing lengths were measured. The hatch date of each brood was recorded directly during routine nest site monitoring, or back‐calculated from measured chick wing lengths using a previously derived relationship (Daunt, 2000). To further validate this calculation, hatch date was both recorded directly and back‐calculated for 283 broods hatched during 2010–2012. The mean difference in estimated hatch date was 1.6 ± 2.4 *SD* days, demonstrating that the two methods give consistent results. Breeding success was recorded as the number of chicks fledged per nest recorded through frequent systematic nest checks throughout the season. Sample sizes for hatch date are smaller than for breeding success because hatch date was not observed or accurately estimable for some broods that failed before ringing.

Shags sometimes attempt to breed at 2 years of age, but then typically have later hatch dates and lower breeding success than birds aged at least 3 years (Aebischer, 1993; Daunt et al., 1999; Potts, Coulson, & Deans, 1980). Subsequently, reproductive performance varies relatively little with age within adults (Daunt et al., 1999). As sample sizes of breeding 2‐year olds were small and our current aim was not to quantify early‐life variation in reproduction or migration, current analyses were restricted to full adults (birds aged 3 years or older), thereby minimising age‐specific variation in reproductive performance. Further consideration of age‐specific variation across our focal sample is provided in Appendix S2.

### 2.4 Statistical analyses

Mark–recapture models fitted to the long‐term Isle of May resighting data show that the probability of resighting a breeding adult during 2010–2012 was 0.98 (S. Burthe, F. Daunt, S. Wanless, J. M. Reid, unpublished data). Further multi‐event models provided no evidence of non‐breeding by experienced adults in these years (breeding probability >0.99, A. M. Lee, F. Daunt, S. Wanless, J. M. Reid, unpublished data). The small amount of breeding season observation failure was primarily attributable to a few pairs that bred in inaccessible locations rather than to any specific life‐history strategy. Mark–recapture models also showed that apparent adult annual survival probabilities spanning winters 2009–2012 were unusually high (≥0.97), reflecting benign winter conditions. As resighting, breeding and survival probabilities were all so high (≥0.97), and individuals were very rarely observed to switch between resident and migrant locations within winters, further mark–recapture models were not necessary to estimate relationships between sex, migratory strategy and reproductive performance across individuals observed in our focal years and winter areas.

Consequently, we first fitted generalised linear mixed models to test whether observed male or female shags were more likely to be classified as migrant or resident in each winter, and hence test for sex‐biased migration across the surveyed areas. Migratory strategy (i.e. resident or migrant) was modelled as a binary‐dependent variable, with additive and interacting fixed effects of winter and sex, and random individual effects to account for non‐independent observations of individuals that were resighted and bred in multiple years and hence appeared multiple times in the dataset.

We then fitted two further sets of generalised linear mixed models to test whether hatch date or breeding success differed between migrants and residents, with hatch date (number of days from 1 April, with Gaussian error structure and identity link) or breeding success (number of chicks fledged, with Poisson error structure and log link) as dependent variables. Models were fitted to data for all three winter–summer periods combined with additive and interacting fixed effects of winter and migratory strategy, and random individual effects to account for multiple observations of individuals (additional models for each year separately are shown in Appendix S3).

The above models were fitted to data for males and females separately because migratory strategies were not always observed for both individuals in a breeding pair. However, as shags are socially monogamous within breeding seasons, hatch date and breeding success presumably partly reflect the properties of male–female pairs rather than either individual entirely independently (e.g. Brommer et al., 2015). To quantify relationships between a pair's reproductive performance and its joint migratory strategy, we fitted further general linear mixed models to data from breeding attempts made by pairs where both individuals were classified as resident or migrant. Random pair effects were fitted to account for multiple observations, along with fixed effects of year and pair strategy, with strategies defined as “resident male, migrant female,” “resident female, migrant male,” “resident–resident” or “migrant–migrant.” Sample sizes were insufficient to test for pair strategy by year interactions.

Likelihood ratio tests between models that did and did not contain effects of migratory strategy were used to test whether estimated effects on reproductive performance differed significantly from zero. Hatch date was not included in models explaining variation in breeding success, thereby testing whether relationships between breeding success and migratory strategy were statistically explained by hatch date, because breeding attempts where hatch date was unknown were biased towards those that failed.

Finally, chi‐squared tests were used to test whether the frequencies of the four pair strategies differed from those expected given random pairing, thereby testing for non‐random pairing with respect to migratory strategy. Expected frequencies were computed from the observed numbers of migratory and resident individuals across all classified males and females.

Analyses were implemented in R 2.15.0 (R Development Core Team 2008), utilising package lme4 (Bates, Maechler, Bolker, & Walker, 2014).

## 3 RESULTS

### 3.1 Data structure

Overall, 439 individual shags that were observed breeding on the Isle of May in summers 2010–2012 had been resighted and hence classified as migrant or resident during the preceding winter, comprising 211 females, 224 males and 4 individuals of unknown sex that were removed from the dataset. The sex ratio of the remaining 435 individuals therefore did not differ from 50:50 (51% males, 49% females, $\chi_{1}^{2}$ = 0.39, *p* = .53). Furthermore, of all individuals that were observed breeding but not resighted during the preceding winter, 51% were male and 49% were female. Together, these data imply that our winter resighting dataset is not sex‐biased.

Of the 435 known‐sex individuals, 278 (64%), 110 (25%) and 47 (11%) were resighted and bred in one, two or three winter–summer periods respectively. Of 207 cases where an individual was resighted in two consecutive winters (involving 157 individuals), there were only 12 cases of apparent migratory strategy switching (six migrant to resident switches and six resident to migrant switches, involving seven males and five females). Strategy switching was therefore rare, and was not directional or sex‐biased.

### 3.2 Male and female migratory strategy

Across all 3 years, 243 of the 435 known‐sex individuals were classified as resident and 192 were classified as migrant. Of these individuals, 124 (52%) residents were male and 119 (62%) migrants were male. [Correction added after online publication on 21 June 2017: “119 (52%) migrants” changed to “119 (62%) migrants”]. Individuals that were classified as migrants were not significantly more likely to be male than female (β = 0.42, *p* = .43), and the interactive effect of sex and year on migratory strategy was not significant (estimated effect −0.13, *p* = .56, Appendix S3). Both sexes were therefore partially migratory, and there was no evidence of substantial or consistent sex‐bias in migratory strategy across the surveyed areas.

### 3.3 Male and female migratory strategy and hatch date

There were 380 known‐sex individuals whose hatch date was observed or estimated in one or more summers during 2010–2012 and that were classified as resident or migrant in the preceding winter, providing 560 observations of hatch date in total. Mean hatch dates (±1 *SD*) were 19 May ± 6.0 days, 12 May ± 9.5 days and 12 May ± 11.4 days in summers 2010, 2011 and 2012 respectively.

Overall, resident males and females hatched their broods significantly earlier than migrant males and females, with estimated mean differences of approximately 6 days in both sexes (Table 1). The year by migratory strategy interactions were not significant (estimated effects: males, β = 0.89, *p* = .56; females β = 0.44, *p* = .82), showing that the difference in hatch date between residents and migrants was consistent across the 3 years (see also Appendix S3).

**Table 1:** Summary statistics and modelled relationships between aspects of reproductive performance (brood hatch date or breeding success) and migratory strategy across male (M) and female (F) shags that bred in one or more summers during 2010, 2011 and 2012 combined. Raw mean (±1 standard deviation) hatch date (days since 1 April) and breeding success (number of chicks fledged) of migrant and resident males and females are shown. β is the model‐estimated effect size for migrants vs. residents (with 95% confidence intervals), and *p* is the probability that the estimated effect could be observed by chance. N_I_ values are the total numbers of individual residents and migrants (not the total number of observations)

	Sex	N_I_ residents	N_I_ migrants	Resident raw mean ±1 *SD*	Migrant raw mean ±1 *SD*	β [95% CI]	*p*
Hatch date	M	116	88	40.8 ± 11.7	47.0 ± 10.2	5.6 [3.3, 7.9]	<.01
	F	104	72	40.4 ± 9.6	46.9 ± 11.0	5.5 [2.6, 8.5]	<.01
Breeding success	M	119	92	2.1 ± 1.1	1.8 ± 1.1	−0.20 [−0.35, −0.04]	.01
	F	124	100	2.0 ± 1.0	1.7 ± 1.1	−0.17 [−0.35, −0.01]	.05

### 3.4 Male and female migratory strategy and breeding success

There were 435 known‐sex individuals whose breeding success was recorded in one or more summers during 2010–2012 and that were classified as resident or migrant in the preceding winter, providing 651 observations of breeding success in total. Individual breeding success varied from 0 to 4 chicks fledged (Appendix S3). Mean (±1 *SD*) breeding success was 2.3 ± 1.1, 2.1 ± 1.1 and 1.6 ± 1.1 chicks in 2010, 2011 and 2012 respectively, implying that environmental conditions for reproduction varied somewhat among the three focal years.

Overall, resident males and females fledged significantly more chicks per year than migrant males and females, with estimated mean differences of approximately 0.2 chicks per year for both sexes (Table 1). The year by migratory strategy interactions were not significant (estimated effects: males, β = −0.08, *p* = .59; females β = −0.08, *p* = .52), again showing that the difference in breeding success between residents and migrants was broadly consistent across the 3 years (see also Appendix S3).

### 3.5 Pair migratory strategy and reproductive performance

There were 75 breeding attempts where both the male and female were classified as resident or migrant based on resightings during the immediately preceding winter, and all four possible “pair migratory strategies” were recorded (Table 2; Appendix S4). The observed frequencies of pair migratory strategy did not differ from those expected given random pairing ($\chi_{3}^{2}$ = 6.1, *p* = .11; Table 2; Appendix S4). Resident and migrant shags were therefore not significantly more or less likely to pair with resident or migrant mates than expected by chance.

**Table 2:** Summary statistics and modelled relationships between brood hatch date and breeding success and pair migratory strategy across shag pairs where both the female and male were classified as resident or migrant. The expected numbers of attempts are the frequencies of pair migratory strategies given random pairing. Raw mean (±1 standard deviation) hatch date and breeding success of pairs comprising migrant or resident males and females are shown. β is the model‐estimated effect size for pair migratory strategy, with 95% confidence intervals. Models that included pair migratory strategy as an explanatory factor fitted significantly better than models without this factor (hatch date, LRT, χ^2^ = 147.0, *p* < .01; breeding success, LRT, χ^2^ = 10.7, *p* = .05)

Male strategy	Female strategy	No. of attempts	Expected no. of attempts	Hatch date		Breeding success	
				Raw mean ±1 *SD*	β [95% CI]	Raw mean ±1 *SD*	β [95% CI]
Resident	Resident	35	30	36.6 ± 7.5	37.2 [34.0, 40.6]	2.3 ± 1.0	0.8 [0.62, 1.05]
Resident	Migrant	14	19	48.4 ± 14.5	48.6 [42.7, 54.5]	1.6 ± 1.1	0.5 [0.07, 0.87]
Migrant	Resident	11	16	41.0 ± 8.5	41.0 [34.8, 47.2]	1.6 ± 1.1	0.5 [0.03, 0.95]
Migrant	Migrant	15	10	45.5 ± 9.0	45.6 [40.4, 50.7]	1.6 ± 1.0	0.5 [0.03, 0.87]

**Figure 2:**
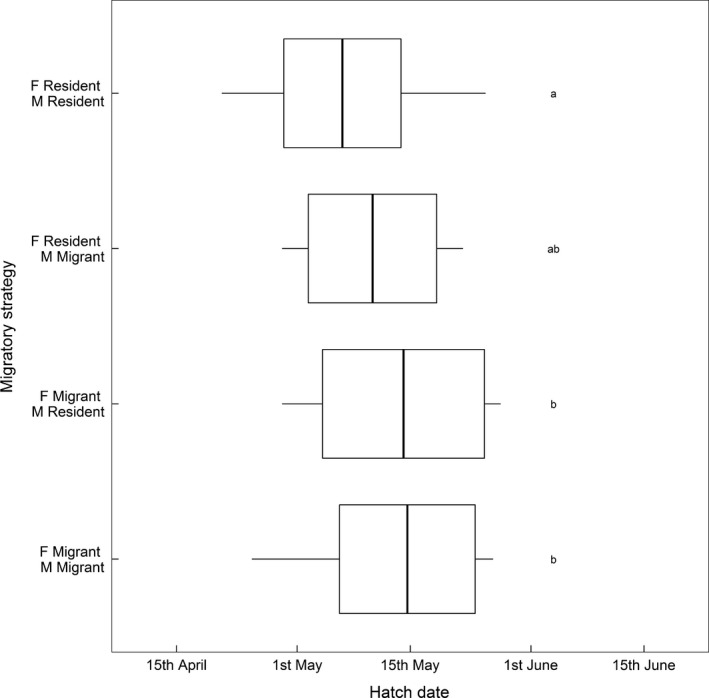
Distributions of hatch dates of breeding attempts made by shag pairs where both the male (M) and female (F) were classified as resident or migrant. Thick bars and boxes show raw mean breeding success ±1 standard deviation, and whiskers demarcate the full range. Lowercase letters indicate significantly different modelled groups

Hatch date was recorded for 64 of 75 breeding attempts where pair migratory strategy was known. “Resident–resident” pairs hatched broods 12 days earlier than “migrant–migrant” pairs on average (Table 2; Figure 2), with mean hatch dates of 6 May and 18 May respectively. There were also differences between pairs with mixed migratory strategies: “resident female, migrant male” pairs hatched broods 7 days earlier than “resident male, migrant female” pairs on average (Table 2; Figure 2).

**Figure 3:**
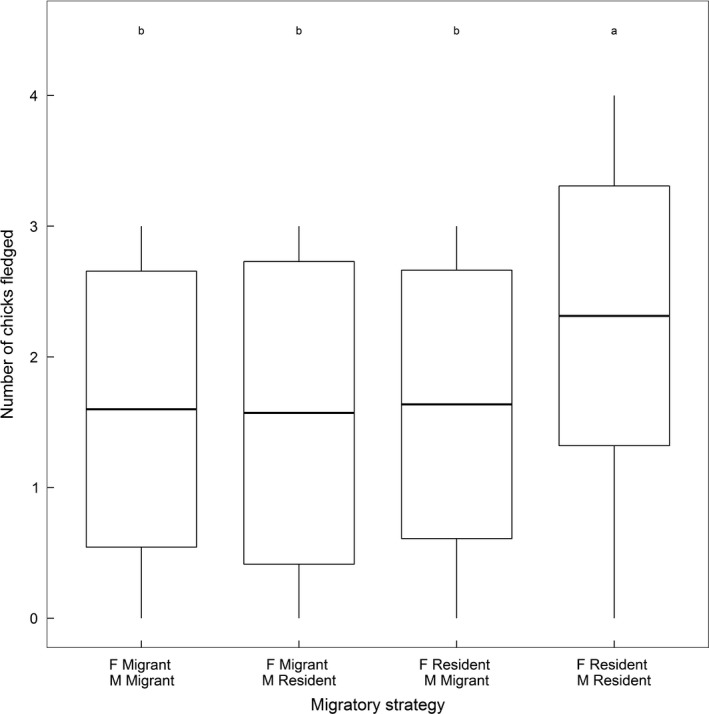
Distributions of breeding success of shag pairs where both the male (M) and female (F) were classified as resident or migrant. Thick bars and boxes show raw mean breeding success ±1 standard deviation, and whiskers demarcate the full range. Lowercase letters indicate significantly different modelled groups

Across all 75 pairs, “resident–resident” pairs also had substantially higher breeding success than pairs where one or both individuals were migrant. Specifically, resident–resident pairs fledged a mean of 2.3 chicks compared to approximately 1.6 chicks for all other pair migratory strategies (Table 2; Figure 3).

## 4 DISCUSSION

Quantifying the degree to which reproductive performance differs between resident and migrant males, females and breeding pairs within any population is central to understanding the ongoing evolution of migratory strategy, and to understanding the potential consequences of partial migration for demographic structure and population dynamics (Chapman et al., 2011; Gillis et al., 2008; Pulido, 2007). We show that, in European shags, both sexes are partially migratory and that resident males and females hatched their broods earlier and fledged more chicks per year than migrant males and females. Moreover, these aspects of reproductive performance varied with the joint migratory strategy of a pair, such that resident–resident pairs hatched their broods earlier, and had higher breeding success than pairs comprising one or two migrants.

### 4.1 Individual migratory strategy and reproductive performance

Resident individual male and female shags fledged ~0.2 more chicks per year than migrant individuals on average, a difference that equates to ~10% of the grand mean breeding success during 2010–2012 of ~2 chicks. Moreover, resident males and females hatched their broods ~6 days earlier than migrant individuals on average. This difference in timing could increase the difference in the contributions of residents vs. migrants to population growth rate beyond that expected solely from their relative breeding success. Chick survival from ringing to recruitment is negatively correlated with hatch date in shags, as in many other bird species (e.g. Arnold, Hatch, & Nisbet, 2006; Lindholm, Gauthier, & Desrochers, 1994). Specifically, shag chicks have a 0.01 increase in recruitment probability for every week's advance in hatch date, and this increase has been attributed primarily to higher post‐fledging survival (Harris et al., 1994). Overall, resident male and female shags will therefore contribute more recruits to the population per year per capita than migrant males and females.

Some previous studies on other systems also showed that residents can have better reproductive performance than migrants (e.g. American dippers, *Cinclus mexicanus*, Gillis et al., 2008; American kestrels, *Falco spaverius*, Anderson et al., 2015; Table S1). Such evidence is consistent with an assumption of evolutionary models of partial migration that residence can be associated with higher reproductive success (Griswold et al., 2010; Kokko, 2011). However, studies of other species did not find such patterns (e.g. Elk, *Cervus canadensis*, Hebblewhite & Merrill, 2011; Skylark, *Alauda arvensis*, Hegemann et al., 2015; Table S1). Furthermore, the few studies that quantified associations between reproductive performance and migratory strategy in both males and females have often found sex‐specific effects (e.g. Snowy plovers, Warriner et al., 1986; Lanyu scops owls, Bai et al., 2012; Table S1). Therefore, in showing that the reproductive performance of resident shags exceeded that of migrants to similar degrees in both sexes, our study adds to the diversity of observed patterns of sex‐specific variation in reproductive performance with migratory strategy, implying that such patterns cannot yet be generalised and likely depend on system‐specific ecology and mechanisms.

### 4.2 Pair migratory strategy and reproductive performance

Across shag breeding attempts where both the male and female were classified as resident or migrant, reproductive performance varied with the pair's combined migratory strategy. Specifically, “resident–resident” shag pairs hatched their broods 12 days earlier than “migrant–migrant” pairs on average, exceeding the difference of 6 days estimated between resident and migrant individuals of each sex independently. In addition, the greatest difference in breeding success was between “resident–resident” pairs and all other pairings; “resident–resident” pairs produced an average of 0.7 chicks per year more than pairs that contained one or two migrants, representing a substantial proportional increase.

Evolutionary and population dynamic models of partial migration often consider single‐sex populations to facilitate model tractability (e.g. Kokko, 2011; Vélez‐Espino, McLaughlin, & Robillard, 2013). However, the assumption that the reproductive performance of resident and migrant individuals of one sex does not vary with the migratory strategies of their mates may be frequently violated in natural systems, particularly in populations with socially persistent breeding pairs. Our study illustrates that an individual's reproductive performance is not necessarily independent of the migratory strategy of its mate. Variation in reproductive performance could therefore be over‐ or under‐estimated in empirical and theoretical studies that consider each sex separately. Quantifying variation in reproductive performance in relation to pair migratory strategies in diverse species and breeding systems may therefore be an important step in understanding the evolutionary maintenance of mixed migratory strategies and resulting population dynamics.

To our knowledge, only one previous study explicitly quantified relationships between reproductive performance and pair migratory strategy. Warkentin et al. (1990) found that merlin pairs containing at least one resident individual hatched broods up to 4 days earlier than “migrant–migrant” pairs. However, merlins and shags showed opposite patterns of reproductive performance in mixed strategy pairs: in contrast to shags, “resident male, migrant female” merlin pairs fledged more chicks than “resident female, migrant male” pairs. This indicates that pair reproductive performance varies more with male migratory strategy than with female strategy in merlins, possibly due to the male's primary contribution to prey capture (Espie, Oliphant, James, Warkentin, & Lieske, 2000). While the shag and merlin studies therefore both indicate that “resident–resident” pairs have highest reproductive success, they highlight that the directions of mixed pair effects may vary among systems, perhaps reflecting species‐ or population‐specific variation in sex roles and ecology.

Dependence of reproductive performance on pair migratory strategy could also potentially drive mate choice among residents and migrants, and create intrinsic frequency‐dependence in the fitness consequences of individual migration vs. residence. However, perhaps surprisingly, there was no evidence of assortative pairing with respect to migratory strategy in shags; residents and migrants were no more or less likely to pair with residents or migrants than expected by chance. This indicates that there is no sympatric reproductive isolation between residents and migrants, and therefore no evidence of underlying genetic structure (e.g. Anderson et al., 2015; Pulido, 2007). The lack of assortative pairing also means that many paired males and females that bred together cannot over‐winter together. Divergent winter conditions occurring in different areas might mean that mates experience different conditions, potentially decoupling reproductive timing the following spring and driving divorce (Gunnarsson et al., 2004). Indeed, shags are much less mate‐faithful across years than many seabirds (Aebischer et al., 1995). However, as “resident–resident” shag pairs had the highest breeding success and pairs with a resident female bred earlier, choice for resident mates might be expected. Future analyses could therefore test whether patterns of divorce and repairing are non‐random with respect to male or female migratory strategy.

### 4.3 Mechanisms and implications

Ultimately, the population dynamic and evolutionary consequences of observed relationships between migratory strategy and aspects of reproductive performance such as hatch date and breeding success will depend on the underlying mechanisms. Relationships could potentially be direct and causal, such that an individual's migratory strategy affects its reproductive performance through one or more non‐exclusive mechanisms. First, time or energy costs of migration movements themselves could directly constrain breeding date or success. However, while the 200–300 km distance between the Isle of May and the surveyed migrant wintering areas is sufficient to constitute a definitive seasonal movement (Figure 1, Grist et al., 2014), shags breeding on the Isle of May typically have a breeding season foraging range of 8–11 km and make 1–4 feeding trips per day (Bogdanova et al., 2014). It therefore seems unlikely that a 200–300 km seasonal migration imposes sufficient energetic cost to directly impact reproductive performance. Moreover, tracking data show that shags can accomplish 200–300 km migratory movements within 1–2 days (S. Wanless, unpublished data), which is substantially less than the observed delay in migrants' hatch dates.

Second, direct effects of migratory strategy on reproductive performance could arise because residents can pre‐emptively occupy high quality nest sites while migrants are away (e.g. Kokko, 2011). Indeed, studies of other shag populations have shown that nest sites associated with higher breeding success are occupied first (Velando & Freire, 2003), although the characteristics that determine these “higher quality” sites vary between colonies (Aebischer, 1985; Potts et al., 1980; Velando & Freire, 2001). However, any such effects might be expected to be stronger in male shags, as males are primarily responsible for nest defence (Snow, 1960), and therefore seem unlikely to explain the higher breeding success of resident females.

Third, the relationships between migratory strategy and reproductive performance observed in both sexes might reflect “carry‐over” effects, where residents might utilise higher quality winter foraging habitat than migrants, and consequently start the breeding season in better condition and/or reach breeding condition earlier (e.g. Harrison, Blount, Inger, Norris, & Bearhop, 2011). Such mechanisms might explain earlier breeding by resident females, and why mixed strategy “resident female, migrant male” pairs hatched their broods earlier than “resident male, migrant female” pairs, as the timing of breeding in shags has been suggested to predominantly reflect female foraging efficiency and associated condition (Daunt et al., 2006).

Alternatively, there might be little or no direct effect of individual migratory strategy on reproductive performance. Instead, the observed associations could arise from, or be exacerbated by, correlated consequences of variation in some underlying trait or state, often conceptualised as “individual quality.” For example, Adriaensen and Dhondt (1990) suggested that migrant robins have lower reproductive performance and survival than residents because less competitive individuals were forced to migrate. However, individual migratory strategy appears not to be a highly flexible condition‐dependent or age‐dependent strategy in individual shags. Individual adults are very highly repeatable in their migratory strategy and location across winters (Grist et al., 2014), and even though individual and population‐wide breeding success varied within and across our three study years, individuals rarely switched migratory strategy. Any state‐dependence underlying observed associations between migratory strategy and breeding success is therefore very unlikely to stem from reverse causality such that breeding failure causes facultative migration (as observed in black‐legged kittiwakes, *Rissa tridactyla*, Bogdanova et al., 2011). Furthermore, mark–recapture models fitted to winter sighting data collected during 2009–2015 and spanning diverse winter conditions show that between‐winter transition probabilities between different wintering areas, and hence between migratory strategies, are generally low for both sub‐adult and adult shags (J. Sturgeon, S. Burthe, F. Daunt, S. Wanless, J. M. Reid, unpublished data). Individuals' migratory strategies therefore appear to be set early and largely remain fixed through life, implying that the observed relationships between adult migratory strategy and reproductive performance cannot be attributable to correlated effects of age (Appendix S2). However, the observed relationships might reflect indirect effects of an underlying fixed state variable that permanently affects both individual migratory strategy and mean reproductive performance. This situation could potentially generate indirect selection on migratory strategy, and generate strong and persistent structured covariation in migratory strategy and reproductive performance at both individual and cohort levels, influencing spatio‐temporal population dynamics (e.g. Lindström & Kokko, 2002; Vindenes et al., 2008).

Lifelong longitudinal data on individual migratory strategy, reproductive performance and survival are ultimately required to evaluate the overall fitness consequences of migratory strategy and hence evaluate population dynamic and evolutionary implications; but such data are not yet available for any partially migratory system (Gaillard, 2013). During our three study years, winter environmental conditions were consistently good, resulting in high annual survival probabilities for adult shags breeding on the Isle of May (0.97–0.98). Consequently, survival probability did not differ between residents and migrants across these winters (J. Burthe, F. Daunt, S. Wanless, J. M. Reid, unpublished data). Over longer time‐scales spanning harsher winter conditions, the relatively high breeding success of residents could potentially be balanced by decreased survival, facilitating evolutionary maintenance of mixed migration strategies (Sanz‐Aguilar et al., 2012). Indeed, resident American dippers had higher breeding success than migrants but lower over‐winter survival (Gillis et al., 2008). Furthermore, our analyses only included migrants that wintered in specific surveyed areas (Figure 1), and migrants that wintered elsewhere might have had higher or lower breeding success. However, the surveyed migrant areas hold relatively large numbers of migrant Isle of May breeders, and hatch date and breeding success did not differ markedly or systematically among migrants observed at different roosts within the surveyed areas (Appendix S5). These data imply that reproductive performance may differ more between residents and migrants than between sets of migrants that move to different destinations. However, estimates of survival and breeding success of migrant and resident individuals over a greater range of environmental and weather conditions and more extensive spatial scales will ultimately be required to understand the associations with overall fitness and the underlying mechanisms, and in particular how mixed strategies are maintained within a single population (Chapman et al., 2011; Gaillard, 2013).

## AUTHORS' CONTRIBUTIONS

H.G., F.D., S.W., S.J.B., M.A.N., M.P.H. and J.M.R. collected the data; H.G. analysed the data; H.G. with F.D., S.W. and J.M.R. wrote the manuscript. All authors contributed critically to the drafts and gave final approval for publication.

## DATA ACCESSIBILITY

The data are available from the Dryad Digital Repository https://doi.org/10.5061/dryad.532j0 (Grist et al., 2017).
