# Supplementary material for: Reproductive performance of resident and migrant males, females and pairs in a partially migratory bird
Source: J Anim Ecol. 2017 Jun 19;86(5):1010–21. doi: 10.1111/1365-2656.12691 (PMC6849534; doi:10.1111/1365-2656.12691)
Supplement: Supplementary file 1 [file JANE-86-1010-s001.docx]

**Table S1**. Studies that related measures of reproductive performance to migratory strategy in partially migratory populations. N is the largest number of individuals (i.e. sample size) analysed within each study. ‘Sex bias’ states which sex is more likely to migrate, or ‘equal’ if both sexes are equally partially migratory. ‘Sex analysed’ states the sexes for which data were analysed. ‘Measure(s)’ states the metrics of reproductive performance considered.

| Species | N | Sex bias | Sex analysed | Measure(s) | Results | References |
| --- | --- | --- | --- | --- | --- | --- |
| American dipper (*Cinclus mexicanus*) | 342 | Equal | Males and females | Hatching date; brood size; nestling condition; fledgling survival | In this ‘breeding season partial migrant’ where residents and migrants breed in different areas, residents bred earlier, had larger broods and were more likely to have second broods than migrants. Chicks produced by residents had better nestling condition and post-fledging survival than chicks produced by migrants. | Mackas *et al*. (2010); Morrissey et al. (2004); Gillis et al. (2008) |
| American kestrels (*Falco spaverius*) | 210 | Equal | Males and females | Laying date | Resident males and females laid eggs earlier in two of the three winters analysed. A female’s migratory strategy predicted her mate’s migratory strategy, i.e. residents were likely to breed with residents. | Anderson *et al*. (2015) |
| Elk (*Cervus canadensis* | 150 | Equal | Females only | Pregnancy rates; calf weights | Resident females had lower pregnancy rates and calf weights than migrant females. | Hebblewhite & Merrill (2011) |
| European robin (*Erithacus rubecula*) | 76 | Female | Males and females in separate studies | Proportion of individuals paired; pairing date | A higher proportion of resident males than migrant males were paired. Resident females paired earlier than migrant females. | Adriaensen & Dhondt (1990); Harper (1985) |
| Giant tortoise (*Testudo gigantea)* | 39 | Equal | Females only | Number of pre-ovulatory follicles | Migrant females had more follicles than resident females. | Swingland *et al*. (1979) |
| Lanyu scops owl (*Otus elegans botelensis*) | 129 | Female | Males and females | Probability of breeding; nesting success; brood size | Resident males were more likely to breed than migrant males, but there was no difference in nesting success or brood size. Resident females were no more likely to breed or have larger brood sizes than migrant females, but had higher nesting success. | Bai, Severinghaus & Philippart (2012) |
| Merlin (*Falco columbarius*) | 118 | Equal | Males and females | Hatch date; brood size | Pairs with >1 resident hatched chicks earlier than migrant-migrant pairs. Pairs with a resident male had higher brood sizes than all other pair combinations. | Warkentin *et al*. (1990) |
| Red-spotted newt (*Notophthalmus viridescens viridescens*) | 110 | Female | Males and females in separate studies | Tail fin height; lay date; number of eggs; larval size | Resident males developed tail fins earlier than migrant males. Resident females produced larger larvae than migrant females, but did not lay earlier or produce more eggs. | Bloch & Grayson (2010); Grayson & McLeod (2009) |
| Skylark (*Alauda arvensis*) | 107 | Equal | Males and females | Number of nestlings, fledglings and recruits | Numbers of nestlings, fledglings and recruits did not differ between residents and migrants. | Hegemann, Marra & Tieleman (2015) |
| Snowy plover (*Charadrius nivosus*) | 43 | Equal | Males and females | Pairing date | Resident males paired earlier than migrant males, but pairing date did not differ between resident and migrant females. | Warriner *et al*. (1986) |
| Spruce grouse (*Canachites canadensis*) | 118 | Female | Females only | Proportion of females breeding | The proportions of migrant and resident females breeding did not differ. | Herzog *&* Keppie (1980) |
| White-ruffed manakin (*Corapipo altera*) | 117 | Equal | Males only | Attracting females | Resident males attract females to lek sites more frequently and for longer durations than migrant males. | Boyle *et al.* (2011) |

**References**

Anderson, A.M., Novak, S.J., Smith, J.F., Steenhof, K., Heath, J.A. (2015) Nesting phenology, mate choice and genetic divergence within a partially migratory population of American Kestrels. *The Auk*, 133, 99-109.

Bai, M., Severinghaus, L.L. & Philippart, M.T. (2012) Mechanisms underlying small-scale partial migration of a subtropical owl. *Behavioral Ecology,* 23, 153-159.

Bloch, A. & Grayson, K. (2010) Reproductive costs of migration for males in a partially migrating, pond-breeding amphibian. *Canadian Journal of Zoology,* 88, 1113-1120.

Boyle, W.A. (2008) Partial migration in birds: tests of three hypotheses in a tropical lekking frugivore. *Journal of Animal Ecology,* 77, 1122-1128.

Gillis, E.A., Green, D.J., Middleton, H.A. & Morrissey, C.A. (2008) Life history correlates of alternative migratory strategies in American Dippers. *Ecology,* 89, 1687-1695.

Grayson, K.L., & Wilbur, H.M. (2009) Sex- and context-dependent migration in a pond-breeding amphibian. *Ecology,* 90, 306-312.

Grayson, K.L. & Mcleod, H.D. (2009) Evaluating the reproductive cost of migration for females in a partially migrating pond‐breeding amphibian. *Journal of Zoology,* 279, 71-77.

Hebblewhite, M. & Merrill, E.H. (2011) Demographic balancing of migrant and resident elk in a partially migratory population through forage–predation tradeoffs. *Oikos,* 120, 1860-1870.

Hegemann, A., Marra, P.M. &Tieleman, B.I. (2015) Causes and consequences of partial migration in a passerine bird. *American Naturalist*, 186, 531-546.

Herzog, P.W. & Keppie, D.M. (1980). Migration in a local population of Spruce Grouse. *Condor*, 366-372.

Mackas, R., Green, D., Whitehorne, I., Fairhurst, E., Middleton, H. & Morrissey, C. (2010) Altitudinal migration in American Dippers (*Cinclus mexicanus*): Do migrants produce higher quality offspring? *Canadian Journal of Zoology,* 88, 369-377.

Morrissey, C.A. (2004). Effect of altitudinal migration within a watershed on the reproductive success of American dippers. *Canadian Journal of Zoology*, 82, 800-807.

Sanz‐Aguilar, A., Bechet, A., Germain, C., Johnson, A.R. & Pradel, R. (2012) To leave or not to leave: survival trade‐offs between different migratory strategies in the greater flamingo. *Journal of Animal Ecology,* 81, 1171-1182.

Swingland, I.R. & Lessells, C.M. (1979) The natural regulation of giant tortoise populations on Aldabra Atoll. Movement polymorphism, reproductive success and mortality. *Journal of Animal Ecology*, 48, 639-654.

Warkentin, I.G., James, P.C. & Oliphant, L.W. (1990). Body morphometrics, age structure, and partial migration of urban Merlins. *The Auk,* 107, 25-34.

Warriner, J.S., Warriner, J.C., Page, G.W. & Stenzel, L.E. (1986) Mating system and reproductive success of a small population of polygamous Snowy Plovers. *Wilson Bulletin*, 98, 15-37.
