# Supplementary material for: Reproductive performance of resident and migrant males, females and pairs in a partially migratory bird
Source: J Anim Ecol. 2017 Jun 19;86(5):1010–21. doi: 10.1111/1365-2656.12691 (PMC6849534; doi:10.1111/1365-2656.12691)
Supplement: Supplementary file 2 [file JANE-86-1010-s002.docx]

**Appendix I: Details of winter surveys for colour-ringed shags conducted during winters 2009-2010, 2010-2011 and 2011-2012.**

Table A1.1. The frequency of resightings of individual adult colour-ringed shags across all surveyed areas in winters 2009-2010, 2010-2011 and 2011-2012.

| Winter | Number of times resighted | | | | |  | |  |  |  | |  |  |  | |  |  | |  | | Total no. of individuals | |
| --- | --- | --- | --- | --- | --- | --- | --- | --- | --- | --- | --- | --- | --- | --- | --- | --- | --- | --- | --- | --- | --- | --- |
|  | 1 | 2 | 3 | 4 | 5 | | 6 | | | | 7 | | | | 8 | | | 9 | | =>10 | |  |
| 2009-2010 | 115 | 44 | 23 | 13 | 6 | | 2 | | | | 3 | | | | 1 | | | 1 | | 3 | | 211 |
| 2010-2011 | 210 | 140 | 60 | 32 | 3 | | 4 | | | | 5 | | | | 5 | | | 1 | | 14 | | 474 |
| 2011-2012 | 248 | 139 | 70 | 45 | 16 | | 21 | | | | 1 | | | | 4 | | | 5 | | 17 | | 566 |

Table A1.2. The number of resightings of adult colour-ringed shags during October to February in winters 2009-2010, 2010-2011 and 2011-2012 across the surveyed migrant and resident areas (see Fig. 1).

|  | Migrant area | | |  | |  | |  | |  | | Resident area | | |  | |  | |  | |  | Total no. of resightings |
| --- | --- | --- | --- | --- | --- | --- | --- | --- | --- | --- | --- | --- | --- | --- | --- | --- | --- | --- | --- | --- | --- | --- |
| Winter | Oct | | Nov | | | Dec | | Jan | | Feb | | Oct | | Nov | | | Dec | | Jan | | Feb |  |
| 2009-2010 | 65 | 28 | | | 42 | | 66 | | 39 | | 84 | | 85 | | | 6 | | 11 | | 4 | | 430 |
| 2010-2011 | 132 | 54 | | | 70 | | 105 | | 78 | | 160 | | 20 | | | 114 | | 25 | | 358 | | 1116 |
| 2011-2012 | 187 | 95 | | | 151 | | 149 | | 79 | | 207 | | 201 | | | 62 | | 293 | | 49 | | 1473 |

Table A1.3. The number of positive survey days per month across 2009-2010, 2010-2011 and 2011-2012. Positive survey days are dates on which ≥1 colour-ringed adult shag was resighted in a resident and/or migrant area.

| Winter | Area | Number of positive survey days | |  | |  | |
| --- | --- | --- | --- | --- | --- | --- | --- |
|  |  | Oct | Nov | Dec | Jan | Feb | Total |
| 2009-2010 | Resident | 8 | 7 | 2 | 4 | 1 | 22 |
|  | Migrant | 12 | 8 | 8 | 9 | 9 | 46 |
| 2010-2011 | Resident | 10 | 4 | 8 | 5 | 5 | 32 |
|  | Migrant | 19 | 11 | 13 | 18 | 9 | 70 |
| 2011-2012 | Resident | 8 | 13 | 6 | 12 | 6 | 45 |
|  | Migrant | 16 | 14 | 17 | 17 | 9 | 73 |
