# Supplementary material for: Reproductive performance of resident and migrant males, females and pairs in a partially migratory bird
Source: J Anim Ecol. 2017 Jun 19;86(5):1010–21. doi: 10.1111/1365-2656.12691 (PMC6849534; doi:10.1111/1365-2656.12691)
Supplement: Supplementary file 3 [file JANE-86-1010-s003.docx]

**Appendix II: Analysis of variation in reproductive performance with age, cohort and migratory strategy**

In general, indirect associations between individual reproductive performance (i.e. hatch date and breeding success) and migratory strategy (i.e. resident versus migrant) could potentially arise if both reproductive performance and migratory strategy vary concordantly, but independently, with age (or some other state variable). For example, older individuals might both show better reproductive performance and be more likely to be resident. Such indirect mechanisms would not necessarily alter the conclusion that there is, ultimately, selection on migratory strategy but would alter the form of direct versus indirect selection and hence alter expected evolutionary and population dynamic outcomes.

Indirect associations between reproductive performance and migratory strategy stemming from age effects can, in principle, be accounted for by modelling effects of age on reproductive performance alongside effects of migratory strategy. However, in practice, distinguishing the effects of age on any phenotype from confounding effects of survival selection (i.e. selective disappearance) is problematic given cross-sectional data on different individuals of diverse ages observed over a short period (rather than longitudinal data on the same individuals observed across different ages, e.g. Lande & Arnold 1983; van de Pol & Verhulst 2006). For example, in the current context, if migrants were more likely to survive any year than residents, then migrants would on average be older than residents at any time of observation and represent older cohorts. If reproductive performance in fact varied with migratory strategy but not directly with age or cohort, then modelling effects of age or cohort would likely capture some of the variance in reproductive performance that is directly due to migratory strategy and indicate spurious effects of age or cohort. Such analyses should consequently be formulated and interpreted cautiously, and in the light of known attributes of the biology of any focal system.

In our study system, previous analyses showed that two year-old shags that attempt to breed have later hatch dates and lower breeding success than older shags, but that there is little age-specific variation in reproductive performance thereafter (Potts, Coulson & Deans 1980; Daunt *et al*. 1999). Consequently, by excluding data from shags that bred aged two years (see main Methods) we minimised the degree to which any associations between migratory strategy and reproductive performance could stem from covariances with age. Furthermore, there is no evidence that migratory strategy varies markedly with individual age. Individual shags are highly repeatable in their migratory strategy across years (see main text and Grist *et al*. 2014), and typically acquire their strategy soon after fledging (i.e. before commencing reproduction, Sturgeon *et al.* unpublished data). It is therefore unlikely that observed associations between reproductive performance and migratory strategy could result primarily from covariation with age in our system. However, we undertook further analyses to examine such effects.

A substantial proportion of the individual shags included in our main analyses were first caught and ringed as breeding adults, meaning that their age (and cohort) were unknown. Therefore to quantify whether age or cohort effects may explain the observed difference in reproductive performance between residents and migrants, the dataset was restricted to individuals ringed as chicks and hence of known cohort and age at observation during our focal study years (2010-2012). We first fitted the same models relating hatch date or breeding success to migratory strategy as were fitted to the full dataset (see main manuscript) to the reduced dataset, thereby verifying whether the same patterns detected across the full dataset were also evident in the reduced known-age dataset. We then fitted further generalised linear (mixed) models to the reduced datasets, with hatch date (number of days from 1^st^ April with Gaussian error structure) or breeding success (number of chicks fledged with Poisson error structure) as dependent variables, and that included age or natal cohort as additional fixed effects (alongside observation year). Age was measured as the number of years between hatching and the observed breeding event and modelled as a continuous variable with linear and quadratic effects. Cohort (i.e. hatch year) was modelled as a categorical fixed effect. Likelihood ratio tests between models that did or did not contain cohort were used to test whether estimated effects differed significantly from zero.

**Data**

Overall, of the 435 known-sex individuals of known migratory strategy that were subsequently recorded breeding in summers 2010-2012, 272 (63%) were ringed as chicks and were therefore of known age and cohort (comprising 144 males and 128 females).

Four cohorts that were represented by fewer than five observed individuals were additionally excluded from the cohort analyses, leaving 13 remaining cohorts. The earliest included cohort was 1996, and the latest 2009.

The age of individuals retained in the dataset ranged from 3 to 21 years for males, and 3 to 18 years for females.

Hatch date

A total of 330 hatch dates were observed or estimated during summers 2010-2012 across 236 individuals that were both classified as resident or migrant and ringed as chicks (comprising 134 males and 102 females).

Across this reduced dataset, resident males hatched their broods on average five days earlier than migrant males (β= 5.2 [95% CI 2.0, 8.3], p <0.01), and resident females hatched their broods on average five days earlier than migrant females (β= 5.4 [95% CI 1.9, 8.8], p <0.01). These estimated effects are very similar to those estimated across the full dataset that included individuals ringed as adults (main text, Table 1).

When cohort was included in the hatch date analysis, resident males still hatched their broods four days earlier than migrant males (β= 3.7, p=0.03), and there was also a significant effect of cohort on hatch date (χ^2^= 21.3, p=0.05). Resident females also hatched their broods four days earlier than migrant females (β= 3.9 p =0.03), and there was a significant effect of cohort on hatch date (χ^2^= 39.2, p<0.01).

When age was included in the hatch date analysis, resident males still hatched their broods four days earlier than migrant males (β= 3.6, p=0.02), and older males hatched their broods earlier than younger males (β = -2.1, p=0.01; the quadratic age term was not significant, β= 0.07, p =0.08). Resident females also hatched their broods four days earlier than migrant females (β= 4.0, p =0.01), and older females hatched their broods earlier than younger females (β = -4.3, p<0.01). The quadratic age term was also significant for females (β= 0.2, p <0.01), suggesting that the cross-sectional relationship between hatch date and age is non-linear in female shags.

Breeding success

A total of 399 observations of breeding success across 272 individuals ringed as chicks were recorded during 2010-2012. Across the reduced dataset, both resident males and females tended to fledge more chicks than migrant males (β= -0.16, p =0.09), and females (β= -0.22, p =0.05) respectively. This difference was not significant for males, and only marginally significant for females. However, the estimated effects were similar to those estimated across the full dataset; slightly smaller for males and slightly larger for females (main text, Table 3). The non-significant effect in males therefore primarily reflects reduced statistical power resulting from excluding observed individuals that had been ringed as adults rather than any substantively different biological effect.

When cohort was included in the breeding success analysis, resident males and females still tended to fledge slightly more chicks than migrant males (β= -0.17, p=0.12), and females (β= -0.12, p=0.33) although the effects were again not significant. However, the cohort effects were also non-significant for both males (χ^2^= 7.7, p=0.80) and females (χ^2^= 14.7, p=0.20).

When age was included in the breeding success analysis, resident males and females again tended to fledge slightly more chicks than migrant males (β= -0.17, p=0.10), and females (β= -0.17, p=0.14); the estimated effects were similar to those estimated across the full dataset (main text table 1), but again not significant due to the reduced statistical power. The linear and quadratic effects of age were also not significant for males (age: β = 0.09, p=0.10, age^2^: β = 0.01, p=0.10), but significant for females (age: β = 0.21, p=0.01, age^2^: β = -0.01, p=0.02).

Conclusions

Overall, our analyses of reduced datasets comprising individual shags that had been ringed as chicks showed that including age or cohort in the models did not substantially alter the size or direction of the estimated effects of migratory strategy on hatch date or breeding success. Further, any covariance attributed to age or cohort in these analyses must be interpreted with caution given that our data are cross-sectional, and that individual shags are rarely observed to switch migratory strategy between years. The estimated age and cohort effects could consequently be spurious consequences of selective disappearance. It is therefore unlikely that the relationships between reproductive performance and migratory strategy described in the main text are substantively caused by age or cohort effects (see also the ‘Mechanisms and implications’ section in the main manuscript Discussion).

References

Aebischer, N.J. (1993) Immediate and delayed effects of a gale in late spring on the breeding of the shag *Phalacrocorax aristotelis*. *Ibis*, 135, 225-232.

Daunt, F., Wanless, S., Harris, M.P., & Monaghan, P. (1999) Experimental evidence that age-specific reproductive success is independent of environmental effects. *Proceedings of the Royal Society of London B: Biological Sciences*, 266, 1489-1493.

Daunt, F., Monaghan, P., Wanless, S., Harris, M.P., & Griffiths, R. (2001) Sons and daughters: age‐specific differences in parental rearing capacities. *Functional Ecology*, 15, 211-216.

Grist, H., Daunt, F., Wanless, S., Nelson, E.J., Harris, M.P., Newell, M., Burthe, S., & Reid, J.M. (2014) Site fidelity and individual variation in winter location in partially migratory European shags. *PloS One*, 9, e98562.

Lande, R., & Arnold, S.J. (1983) The measurement of selection on correlated characters. *Evolution*, 37, 1210-1226.

Potts, G.R., Coulson, J.C., & Deans, I.R. (1980) Population dynamics and breeding success of the shag, *Phalacrocorax aristotelis*, on the Farne Islands, Northumberland. *Journal of Animal Ecology*, 49, 465-484.

van de Pol, M., & Verhulst, S. (2006) Age‐dependent traits: a new statistical model to separate within‐and between‐individual effects. *American Naturalist*, 167, 766-773.
