# Supplementary material for: Reproductive performance of resident and migrant males, females and pairs in a partially migratory bird
Source: J Anim Ecol. 2017 Jun 19;86(5):1010–21. doi: 10.1111/1365-2656.12691 (PMC6849534; doi:10.1111/1365-2656.12691)
Supplement: Supplementary file 4 [file JANE-86-1010-s004.docx]

**Appendix III: Relationship between migratory strategy and reproductive performance in shags across individual years 2009-2012.**

The analyses relating migratory strategy to sex and reproductive performance presented in the main text utilised all data from the full study period 2009-2012. Here, we provide more detailed descriptive statistics and analyses for each consecutive winter-summer period 2009-2010, 2010-2011 and 2011-2012 separately.

**Data structure in each year**

Overall, 825, 1010 and 1200 individual colour-ringed shags were observed breeding on the Isle of May in summers 2010, 2011 and 2012 respectively. Totals of 305, 808 and 1025 resightings of these adults were recorded across the winter survey areas (main text, Fig. 1) during the winter immediately preceding each summer (i.e. 2009-2010, 2010-2011 and 2011-2012 respectively). These resightings allowed totals of 112, 254 and 285 individuals to be classified as migrant or resident in the three winters following the strict criteria, comprising 14%, 25% and 24% of all individuals observed breeding in the subsequent summer.

Of the individuals retained in the dataset, 39 (35%), 153 (60%) and 161 (56%) were classified as residents in winters 2009-2010, 2010-2011 and 2011-2012 respectively (Table A3.1). This among-year variation in the proportions of individuals that were classified as residents rather than migrants reflects among-year variation in the spatio-temporal distribution of resighting surveys, not necessarily among-year variation in population-wide migratory strategy (Appendix I).

**Male & female migratory strategy in each year**

Of the known-sex individuals that were classified as resident or migrant, 75 (68%), 129 (51%) and 149 (53%) were males in the three winters respectively (Table A3.1).

In total, 71%, 47% and 54% of known-sex residents were male in the three winters respectively, and 65%, 57% and 52% of known-sex migrants were male (Table A3.1). Individuals that were classified as migrants were not significantly more likely to be male than female in any single winter (Table A3.1).

**Table A3.1.** Numbers of individual adult male (M) and female (F) shags classified as resident or migrant in winters 2009-2010, 2010-2011 and 2011-2012. ‘% residents’ gives the percentage of individuals of each sex that was classified as resident. ‘% male’, ‘% residents male’ and ‘% migrants male’ respectively give the percentages of all known-sex individuals, and of known-sex individuals classified as residents and migrants, that were male. Individuals of unknown sex comprised 1 migrant in 2009-2010, 2 residents in 2010-2011 and 1 resident in 2011-2012. β is the binomial model estimate for the effect of sex on migratory strategy (±1 standard error) and p is the probability that the estimated effect could be observed by chance.

| Winter | Sex | No. of residents | No. of migrants | Total | % residents | %  male | % residents male | % migrants male | β ± 1SE | p |
| --- | --- | --- | --- | --- | --- | --- | --- | --- | --- | --- |
| 2009-2010 | M | 28 | 47 | 75 | 37 | 68 | 71 | 65 | -0.30 ± 0.43 | 0.48 |
|  | F | 11 | 25 | 36 | 31 |  |  |  |  |  |
| 2010-2011 | M | 71 | 58 | 129 | 55 | 51 | 47 | 57 | 0.41 ± 0.26 | 0.11 |
|  | F | 80 | 43 | 123 | 65 |  |  |  |  |  |
| 2011-2012 | M | 86 | 63 | 149 | 58 | 53 | 54 | 52 | -0.08 ± 0.24 | 0.73 |
|  | F | 74 | 59 | 133 | 56 |  |  |  |  |  |

### Male & female migratory strategy and hatch date

There were 100, 217 and 243 known sex individuals that were classified as resident or migrant in winters 2009-2010, 2010-2011 and 2011-2012 whose hatch date was observed or estimated in summers 2010, 2011 and 2012 respectively. Mean hatch dates (±1SD) were 19 May ± 6.0 days, 12 May ± 9.5 days and 12 May ± 11.4 days in summers 2010, 2011 and 2012 respectively.

Table A3.2. Summary statistics and modelled relationships between brood hatch date and migratory strategy of male (M) and female (F) shags that bred in summers 2010, 2011 and 2012. Raw mean (±1 standard deviation) hatch dates of broods produced by migrant and resident males and females are expressed as days since 1st April. β is the model-estimated effect size for migrants versus residents (with 95% confidence intervals), and p is the probability that the estimated effect could be observed by chance.

| Summer | Sex | No. of residents | No. of migrants | Resident raw mean ±1SD | Migrant  raw mean ±1SD | β [95%CI] | p |
| --- | --- | --- | --- | --- | --- | --- | --- |
| 2010 | M | 26 | 40 | 47.2±5.9 | 50.3±6.0 | 3.1 [0.2, 6.0] | 0.04 |
|  | F | 10 | 23 | 47.3±6.4 | 48.2±6.4 | 0.9 [-3.8, 5.6] | 0.73 |
| 2011 | M | 63 | 47 | 39.9±8.6 | 44.9±9.2 | 4.9 [2.0, 7.9] | <0.01 |
|  | F | 69 | 26 | 37.8±11.8 | 44.6±11.7 | 6.8 [1.4, 12.2] | 0.01 |
| 2012 | M | 74 | 48 | 41.1±11.2 | 46.6±14.1 | 5.5 [1.4, 9.7] | 0.01 |
|  | F | 61 | 41 | 38.7±11.4 | 46.8±10.2 | 8.1 [3.0, 13.2] | <0.01 |

Resident male shags hatched chicks significantly earlier than migrant males in all three years, with mean differences of approximately 5 days in 2011 and 2012 and 3 days in 2010 (Table A3.2). Similarly, resident female shags hatched chicks significantly earlier than migrant females in 2011 and 2012, with mean differences of approximately 6 and 8 days respectively (Table A3.2). Hatch date did not differ between resident and migrant females in 2010, but sample sizes were small (Table A3.2).

### Male & female migratory strategy and breeding success

There were 112, 254 and 285 known-sex individuals that were classified as resident or migrant in winters 2009-2010, 2010-2011 and 2011-2012, and whose breeding success was recorded in summers 2010, 2011 and 2012 respectively. Mean (±1SD) breeding success was 2.3±1.1, 2.1±1.1 and 1.6±1.1 chicks in 2010, 2011 and 2012 respectively, and individual breeding success ranged from 0-4 chicks fledged across all three years (Table A3.3).

**Table A3.3.** Raw breeding success of individual shags across summers 2010-2012. Breeding success is the number of chicks fledged.

| Summer | Breeding success | | | | |
| --- | --- | --- | --- | --- | --- |
|  | 0 | 1 | 2 | 3 | 4 |
| 2010 | 9 | 15 | 28 | 56 | 4 |
| 2011 | 32 | 34 | 77 | 107 | 4 |
| 2012 | 55 | 70 | 98 | 61 | 1 |

In general, resident male and female shags tended to fledge slightly more chicks than migrant males and females in each of the three years. The estimated differences spanned approximately 0.1-0.3 chicks per year, and were significantly greater than zero for males in 2012 (Table A3.4). The only exception was that resident females tended to fledge fewer chicks than migrant females in 2010, but sample sizes were small and the estimated effect did not differ significantly from zero (Table A3.4).

Table A3.4. Summary statistics and modelled relationships between breeding success and migratory strategy of male (M) and female (F) shags that bred in summers 2010, 2011 and 2012. Raw mean (±1 standard deviation) breeding success of migrant and resident males and females are shown. β is the model-estimated effect size for migrants versus residents (with 95% confidence intervals), and p is the probability that the estimated effect could be observed by chance.

| Summer | Sex | No. residents | No. migrants | Resident raw mean ±1SD | Migrant raw mean ±1SD | β  [95%CI] | p |
| --- | --- | --- | --- | --- | --- | --- | --- |
| 2010 | M | 28 | 47 | 2.4±1.1 | 2.2±1.0 | -0.11 [-0.42, 0.20] | 0.47 |
|  | F | 11 | 25 | 2.1±0.9 | 2.4±1.0 | 0.12 [-0.35, 0.62] | 0.62 |
| 2011 | M | 71 | 58 | 2.2±1.1 | 1.8±1.1 | -0.17 [-0.42, 0.08] | 0.18 |
|  | F | 80 | 43 | 2.3±1.0 | 1.8±1.1 | -0.22 [-0.49, -0.04] | 0.10 |
| 2012 | M | 86 | 63 | 1.9±1.0 | 1.4±1.0 | -0.28 [-0.55, -0.03] | 0.03 |
|  | F | 74 | 59 | 1.6±1.0 | 1.3±1.0 | -0.22 [-0.51, 0.07] | 0.14 |
