# Supplementary material for: Reproductive performance of resident and migrant males, females and pairs in a partially migratory bird
Source: J Anim Ecol. 2017 Jun 19;86(5):1010–21. doi: 10.1111/1365-2656.12691 (PMC6849534; doi:10.1111/1365-2656.12691)
Supplement: Supplementary file 5 [file JANE-86-1010-s005.docx]

**Appendix IV: Details of estimates of assortative pairing between male and female colour-ringed shags with different migratory strategies.**

There were 112, 254 and 285 known sex adult colour-ringed shags that were classified as resident or migrant in winters 2009-2010, 2010-2011 and 2011-2012 respectively, whose brood hatch date was observed or estimated on the Isle of May in summers 2010, 2011 and 2012 respectively. Of these individuals, there were 75 breeding attempts where both the male and female were classified as resident or migrant based on resightings during the immediately preceding winter: 7, 31 and 37 in 2010, 2011 and 2012 respectively (Table A4.1).

These attempts involved 66 individual females and 65 individual males, of which 57 females and 55 males bred in one year, and 9 females and 10 males bred in two years. They included 50 attempts made by 50 discrete female-male pairs that bred with each other once during the focal study years, 10 attempts made by 5 discrete pairs that bred with each other twice, and 15 attempts made by pairs where one or both individuals also bred with a different mate within the dataset.

A chi-squared test was used to test whether the frequency of pair migratory strategies differed from that expected by chance. Data were pooled across years because sample sizes were insufficient to consider each year separately. The expected values were calculated by dividing the total number of individuals of each sex observed by the number of that sex observed of each migratory strategy, and then multiplying the probabilities for the relevant male and female strategies. For example, the expected number of migrant-migrant pairs was calculated by dividing the total number of males observed by the total number of male migrants observed, dividing the total number of females observed by the total number of female migrants observed, and multiplying these two values together.

The observed frequencies of pair migratory status did not differ from those expected given random pairing ($\chi_{3}^{2}$ = 6.1, p = 0.11). Resident and migrant shags were therefore no more or less likely to pair with resident or migrant mates than expected by chance.

**Table A4.1** The number of each type of migratory pairing (‘migrant-migrant’, ‘resident male, migrant female’, ‘resident female, migrant male’, ‘resident resident’) of colour-ringed shags observed breeding on the Isle of May during summers 2010, 2011 and 2012. ‘F’ and ‘M’ denote female and male respectively.

| Summer | F Migrant | F Resident | F Migrant | F Resident | Total |
| --- | --- | --- | --- | --- | --- |
|  | M Migrant | M Migrant | M Resident | M Resident |  |
| 2010 | 4 | 0 | 1 | 2 | 7 |
| 2011 | 3 | 7 | 5 | 16 | 31 |
| 2012 | 8 | 4 | 8 | 17 | 37 |
| All years | 15 | 11 | 14 | 35 | 75 |
