# Supplementary material for: Reproductive performance of resident and migrant males, females and pairs in a partially migratory bird
Source: J Anim Ecol. 2017 Jun 19;86(5):1010–21. doi: 10.1111/1365-2656.12691 (PMC6849534; doi:10.1111/1365-2656.12691)
Supplement: Supplementary file 6 [file JANE-86-1010-s006.docx]

**Appendix V: Analysis of variation in hatch date and breeding success between four focal wintering areas of colour-ringed shags.**

Our primary aim was to test whether reproductive performance differed between individuals that remained resident at the focal breeding colony and individuals that migrated to focal winter areas located ca. 200-300km north. However, to further investigate whether reproductive performance differed between shags that wintered in different geographical areas within the overall focal areas, four wintering areas were defined (Fig. A5.1). Since individual shags make small-scale movements between day and night roosts within a restricted geographical range, a wintering area was defined as a group of known roost sites within <15km (Fig. A5.1).

To quantify variation in reproductive performance between wintering areas, separate analyses were conducted with hatch date and breeding success as dependent variables. All years were pooled using a mixed effects model, with fixed effects of wintering area (areas 1-4, Fig. A5.1) and year, and random individual effects. The overall significance of the relationship between hatch date or breeding success and wintering area was calculated by comparing models with or without wintering area as an explanatory factor using likelihood ratio tests. The significance of differences between wintering areas was calculated using a Tukey post-hoc test.


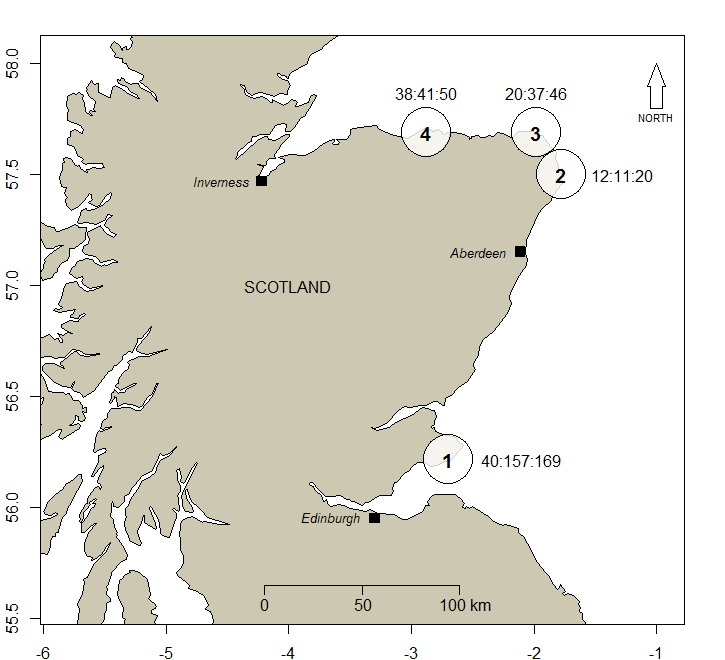


Figure A5.1. Wintering areas of colour-ringed shags breeding on the Isle of May in summers 2010, 2011 and 2012. Figures show the total number of individual shags (of both sexes) that were assigned to each wintering area (1-4) during winters 2009-2010, 2010-2011 and 2011-2012 respectively, that were recorded breeding on the Isle of May in the subsequent summer. Area 1 encompasses the Isle of May, and hence holds resident shags.

### **Sex-specific wintering area and hatch date**

Overall, mean hatch date varied significantly among individuals wintering in different areas in both males (LRT: χ^2^= 28.3, p<0.01, Fig A5.2) and females (χ^2^= 18.1, p<0.01; Fig A5.2) Males and females wintering in Area 1 (i.e. residents) hatched broods a mean of 3-7 days and 4-9 days earlier than males and females wintering in the other three areas (i.e. migrants, Fig A5.1; Fig A5.2; Table A5.1). There was no pattern of later hatch date with increasing distance of wintering area from the Isle of May (e.g. from areas 2-4, Fig. A5.1).


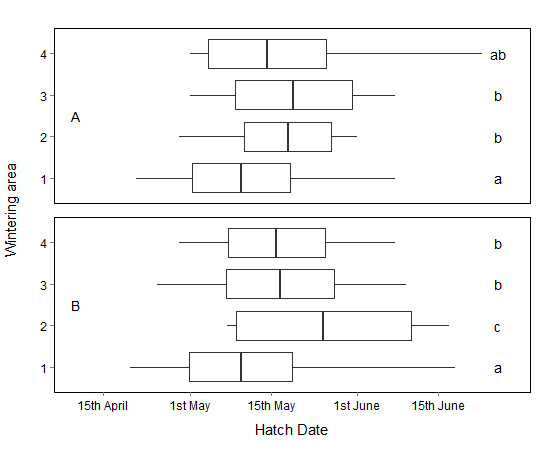


Figure A5.2. Hatch date of broods produced on the Isle of May in summers 2010-2012 by (A) female and (B) male colour-ringed shags that wintered in different areas. Individuals wintering in area 1 are resident, and individuals wintering in areas 2-4 are migrant. Areas are numbered with increasing distance from the breeding colony on Isle of May. Thick bars and boxes show raw mean hatch date ± 1 standard deviation, and whiskers demarcate the full range. Lowercase letters indicate significantly different modelled groups.

Table A5.1. Summary statistics and modelled relationships between brood hatch date and wintering area of male (M) and female (F) shags that bred in summers 2010-2012. Raw mean hatch dates of broods are expressed as days since 1st April. β is the model-estimated effect size relative to zero (with 95% confidence intervals). Individuals wintering in area 1 are resident, and individuals wintering in areas 2-4 are migrant.

| Wintering area | Sex | No. individuals | No. breeding attempts | Raw mean hatch date | β [95% CI] |
| --- | --- | --- | --- | --- | --- |
| 1 | M | 122 | 167 | 40 | 46.4 [43.6, 49.3] |
|  | F | 111 | 142 | 40 | 44.1 [39.8, 48.3] |
| 2 | M | 5 | 7 | 56 | 62.8 [54.5, 71.0] |
|  | F | 21 | 28 | 49 | 51.3 [46.1, 56.5] |
| 3 | M | 41 | 60 | 47 | 52.1 [48.7, 55.6] |
|  | F | 18 | 19 | 50 | 53.1 [47.0, 59.0] |
| 4 | M | 40 | 59 | 46 | 51.2 [47.9, 54.5] |
|  | F | 26 | 36 | 45 | 47.6 43.1, 52.2] |

### **Sex-specific wintering area and breeding success**

The mean number of chicks fledged differed significantly among individuals that wintered in different areas for both males (LRT: χ^2^= 14.3, p<0.01, Fig. A5.3, Table A5.2) and females (χ^2^= 10.2, p=0.02; Fig. A5.3, Table A5.2). Males and females that wintered in the Isle of May area (Fig. A5.1) fledged the most chicks (Fig. A5.3). Breeding success did not differ between males and females that migrated to different areas, and there was no consistent pattern of decreasing breeding success with increasing distance from the Isle of May (i.e. between areas 2-4; Fig. A5.1).


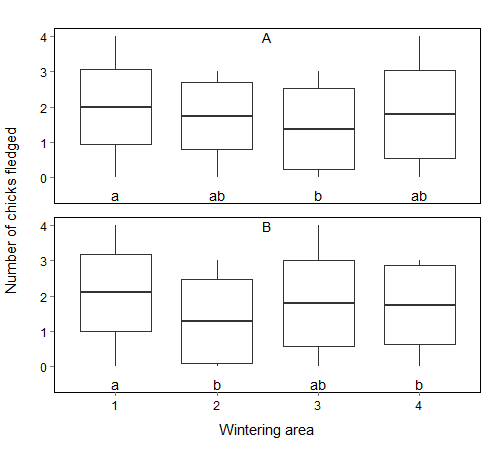


Figure A5.3. Breeding success (i.e. number of chicks fledged) on the Isle of May in summers 2010 - 2012 of (A) female and (B) male colour-ringed shags that wintered in different areas. Individuals wintering in area 1 are resident, and individuals wintering in areas 2-4 are migrant (Fig. A5.1). Thick bars and boxes show raw mean hatch date ± 1 standard deviation, and whiskers demarcate the full range. Lowercase letters indicate significantly different modelled groups.

Table A5.2. Summary statistics and modelled relationships between breeding success and wintering area of male (M) and female (F) shags that bred in summers 2010-2012. Raw mean breeding success is expressed as the number of chicks fledged successfully. β is the model-estimated effect size (with 95% confidence intervals). Individuals wintering in area 1 are resident, and individuals wintering in areas 2-4 are migrant.

| Wintering area | Sex | No. individuals | No. breeding attempts | Raw mean breeding success | β [95% CI] |
| --- | --- | --- | --- | --- | --- |
| 1 | M | 137 | 195 | 2.1 | 0.94 [0.76, 1.11] |
|  | F | 128 | 168 | 2.0 | 0.96 [0.71, 1.21] |
| 2 | M | 8 | 11 | 1.3 | 0.39 [-0.14, 0.92] |
|  | F | 23 | 32 | 1.8 | 0.8 [0.48, 1.11] |
| 3 | M | 46 | 76 | 1.8 | 0.76 [0.54, 0.97] |
|  | F | 25 | 27 | 1.4 | 0.60 [0.21, 0.99] |
| 4 | M | 47 | 77 | 1.7 | 0.71 [0.51, 0.92] |
|  | F | 36 | 49 | 1.8 | 0.79 [0.53, 1.05] |

**Conclusions**

These analyses confirm that in both sexes residents hatched their broods consistently earlier than migrants and had higher breeding success. However, we found no consistent variation in reproductive performance between migrants that moved to different wintering areas. Sample sizes for Areas 2-4 were small and thus our power to detect differences in reproductive performance between the three migrant areas is fairly low. Future analyses of larger datasets might consequently reveal differences which we cannot currently detect.
